# Dynamic extraction coupled on-line to liquid chromatography with a parallel sampling interface—a proof of concept for monitoring extraction kinetics

**DOI:** 10.1007/s00216-019-01849-4

**Published:** 2019-05-06

**Authors:** Mingzhe Sun, Said Al-hamimi, Margareta Sandahl, Charlotta Turner

**Affiliations:** Department of Chemistry, Centre for Analysis and Synthesis, Lund University, P.O. Box 124, 221 00 Lund, Sweden

**Keywords:** Curcuminoids, Dynamic extraction, Liquid chromatography, On-line hyphenation, Parallel sampling, Turmeric powder

## Abstract

On-line hyphenation of extraction with chromatography has been explored in several different types of combinations. However, monitoring the complete process of a dynamic, continuous-flow extraction is not possible with any hyphenated system reported so far. The current work demonstrates that this challenging task can be effectively fulfilled by using a parallel sampling interface, which mimics the concept of comprehensive two-dimensional chromatography. In this study, pressurised hot water extraction was coupled on-line with ultra-high-performance liquid chromatography. The set-up was utilised in a kinetic study of dynamic pressurised hot water extraction of curcuminoids from turmeric powder. Compound-specific extraction curves were obtained, which clearly indicated the rate-limiting factors of the extraction processes under different conditions. Additionally, thermal degradation of curcumin during the extraction could also be demonstrated in some of the extractions.

**Electronic supplementary material:**

The online version of this article (10.1007/s00216-019-01849-4) contains supplementary material, which is available to authorized users.

## Introduction

As an important tool for both sample preparation and product purification, extraction techniques have been widely studied and utilised for many decades. Extraction can be performed in two different modes: static (batch) mode and dynamic (continuous flow) mode. Whilst the static mode generally requires simpler instrumentation and lower consumption of solvent, the dynamic process holds the advantage of enhanced mass transfer, faster kinetics and minimised compound degradation when the extraction is carried out under relatively harsh conditions [1]. In dynamic extraction processes of samples containing one or many major components, the compounds can be extracted at different times and different speeds, and a different final yield is achieved depending on the specific extraction technique and conditions. In order to visualise the extraction profile including all compounds extracted, an on-line detector is usually added at the end of the extraction flow path [2, 3]. Chemometric deconvolution of chromatographic data has been a useful tool in handling signals from co-eluting compounds [4]. This concept has also been applied to resolving extraction curves obtained from on-line detectors into compound or compound class-specific profiles [5]. One main limitation of the curve resolution approach is that analytes cannot have very similar spectra [5]. Hence, to further understand the kinetics of a dynamic extraction process or to obtain qualitative and quantitative information about the extract, chromatographic analysis is still an indispensable step of a complete work flow in most cases [6].

In most cases, chromatographic analysis in an extraction study is performed in two main modes: on-line or off-line. The main advantage of off-line analysis is the simple extract collection, as no special interface is required other than a container for extract collection. Off-line analysis, however, can be time-consuming and suffer from compound loss or degradation [7]. With a well-designed interface, on-line analysis can, to a large extent, eliminate the random errors in off-line collection and subsequent sample preparation procedures, as well as shorten the total run time. There have been several studies in the literature describing the hyphenation of different extraction techniques with on-line chromatographic analysis [8–12]. In most of these applications, the interface is a switching valve consisting of two positions. A loading module is installed in one of the positions for extract collection, and in the other position, a short capillary connection allows the mobile phase of the chromatography to flow through whilst no analysis is taking place. When a selected fraction of the extract is collected, the valve is switched to allow the collected extract to be flushed by the chromatography mobile phase into the analytical column for analysis. This type of set-up can be very useful for the analysis of one or multiple fractions during the extraction. However, since all systems reported so far only utilise one loading module on the interface, continuous monitoring of a real dynamic extraction process is not possible [8, 13]. This is because the extraction process must be temporarily put in static mode to avoid losing fractions of unanalysed extract when the loading module is switched off the extraction path to transfer the collected extract to the chromatographic column.

Our hypothesis is that this drawback can be resolved by on-line coupling of an extraction process with a chromatography system with an interface consisting of two parallel loading modules. The operation of this type of system resembles that of an on-line two-dimensional liquid chromatography (2D-LC) system. When 2D-LC is run in comprehensive mode, the first-dimension eluent is divided into a number of continual segments of the same time length that are called modulations. All modulations are sequentially transferred to the second dimension for fast separations. With a similar working principle, the proposed extraction–chromatography system can potentially allow the dynamic extraction flow to be collected by one sampling module whilst the other parallel sampling module is switched to the analytical flow for analysis. This would enable an on-line monitoring of specific extraction profiles of certain compounds or compound classes during a dynamic extraction process, without the need for sophisticated mathematical deconvolutions. So far, the usefulness of coupling a dynamic process with chromatography using the parallel sampling interface has only been touched upon in the specific case of monitoring drug release of a single compound [14].

To the best of our knowledge, parallel sampling has never been utilised in the on-line hyphenation of extraction with chromatography. In the present study, continuous-flow pressurised hot water extraction (PHWE) was hyphenated with ultra-high-performance liquid chromatography (UHPLC) with an interface consisting of two parallel sampling loops. As a proof of concept, this hyphenated PHWE-UHPLC system was applied for the extraction kinetic study of three major curcuminoids from turmeric powder, where the advantages and limitations of the system were also discussed.

## Materials and methods

### Chemicals

Three curcuminoids, curcumin, demethoxycurcumin and bisdemethoxycurcumin, were purchased from Sigma Chemical Co. (St. Louis, MO, USA). Turmeric powder was purchased from a local supermarket. Ethanol (99.7%) was obtained from Solveco (Rosersberg, Sweden). Acetonitrile was obtained from Scharlau (Barcelona, Spain). All organic solvents were of HPLC grade or above. All water used was from a Milli-Q water purification system with a UV unit.

### Apparatus

**Fig. 1:**
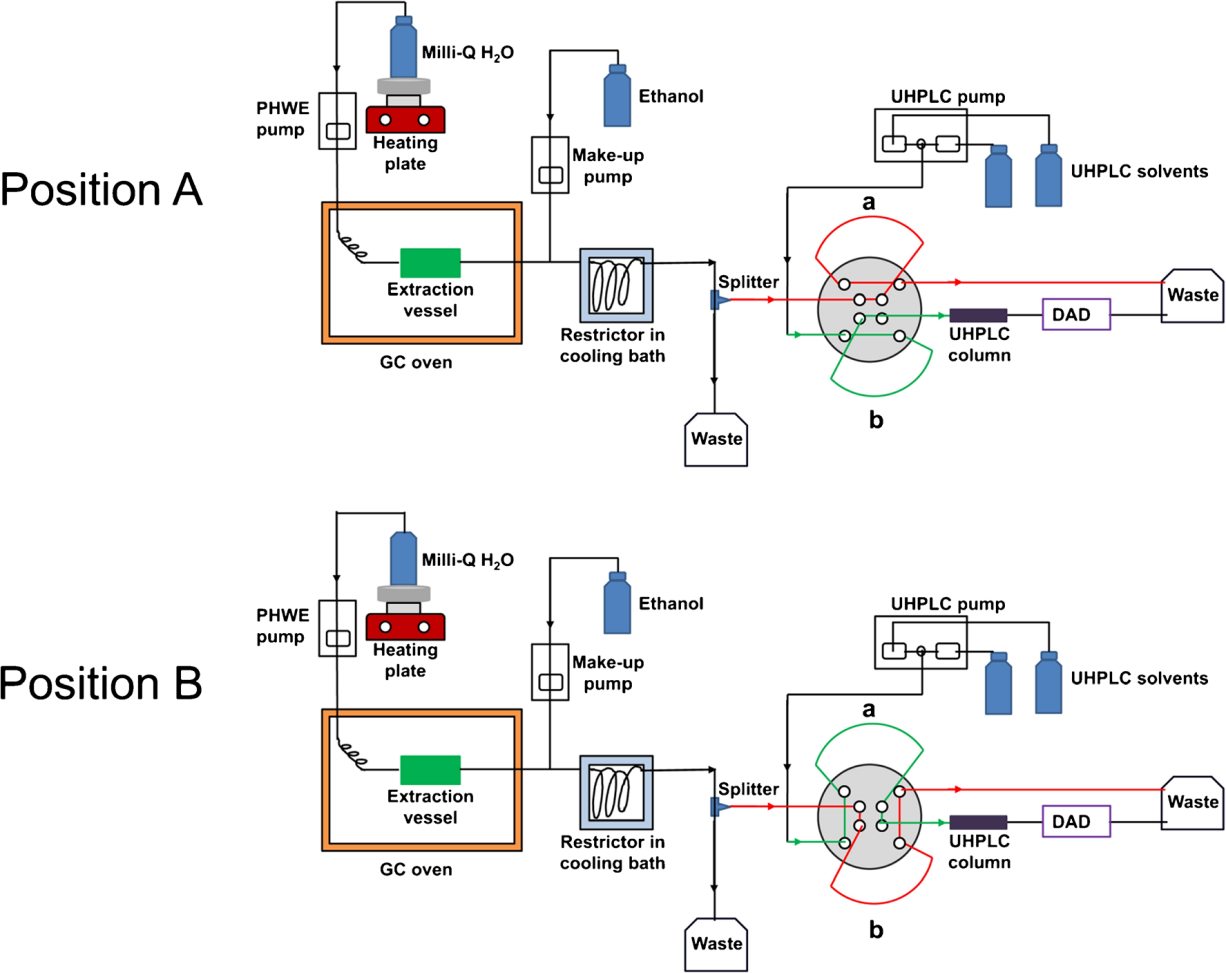
Scheme of the PHWE-UHPLC system

The experiments were performed using a home-built PHWE-UHPLC system. The PHWE system used is similar to one previously described with slight modifications [15]. Water was pre-heated to 70 °C before being pumped into a GC oven held at different temperatures under which the extraction took place. The GC oven contained the extraction cell and a long pre-heating coil installed before the inlet of the extraction cell. The volume of the extraction vessel used was 3 mL. An additional post-extraction make-up ethanol flow was mixed with the H_2_O extract before cooling in order to eliminate any precipitation occurring during the cooling process. The UHPLC part consisted of a degasser (G4225A, Agilent Technologies), a binary pump (G4220A, Agilent Technologies), a thermostated column compartment (G1316C, Agilent Technologies) and a diode array detector (G4212B, Agilent Technologies). As for the interface coupling PHWE with UHPLC, a flexible cube module was used (G4227, Agilent Technologies), in which an 8-port-2-position switching valve (5067–4214, Agilent Technologies) was installed with two identical 60-μL collection loops. A flow splitter was installed between the outlet of the PHWE and the interface (600-PO10-06, Supelco Analytical). For each specific PHWE flow rate after the cooling compartment, a specific split ratio (set by the adjustable flow splitter) was used to allow the collection loop to be filled in one analysis time. The splitting ratios provided by the splitter were verified by collecting the two splitting flows and weighing. The scheme of the PHWE-UHPLC system is shown in Fig. 1. When the interface was at position A, collection loop a is responsible for collecting the fraction of the PHWE extract, whilst the collected extract in loop b is being flushed by the UHPLC flow into the column for analysis. After one analysis time, the interface switches to position B, where the roles of loops a and b also switch. An Agilent Openlab CDS Chemstation C.01.07 software was used for system control. Agilent Chemstation software was used for data processing.

### Column screening and method tuning of the liquid chromatography part

The UHPLC part was connected to the auto-sampler and used as stand-alone UHPLC for column screening and method tuning. Four different columns of the same dimensions but different selectivities available in the lab were screened for the separation of major compounds in turmeric PHWE extract: Agilent Poroshell 120 Eclipse-Cyano (EC-CN, 4.6 × 50 mm, 2.7 μm); Agilent Poroshell 120 Eclipse-C18 (EC-C18, 4.6 × 50 mm, 2.7 μm); Agilent Poroshell 120 Pentafluorophenyl (PFP, 4.6 × 50 mm, 2.7 μm) and Agilent Poroshell 120 Phenyl-hexyl (P-H, 4.6 × 50 mm, 2.7 μm). An extraction of turmeric powder was performed with the same PHWE part in the on-line system. Milli-Q water was pre-heated to 70 °C before being pumped as an extraction solvent. The extraction vessel was heated inside the GC oven to the set temperature and maintained at the temperature for 1 min after the sample was loaded. The dynamic extraction was then started at 90 °C with a water flow rate of 0.5 mL/min and 0.1 mL/min ethanol make-up flow for 1 h. The extract was collected in a 50-mL tube and placed under gentle nitrogen flow in dark for solvent evaporation overnight. The extract after solvent removal was then re-dissolved in ethanol and was further used for screening and optimization experiments. The injection volume was 5 μL. The separation was monitored with DAD at 280 nm and 430 nm with a 20-Hz sampling frequency. The column temperature was set at 30 °C. The flow rate was set at 2.5 mL/min. The mobile phase consisted of A, water (0.3% acetic acid) and B, acetonitrile (0.3% acetic acid). The gradient started with 20% B and then ramp up to 90% in 1.5 min. Then, the gradient was maintained at 90% for 0.5 min before decreasing to the starting composition. The identification of the three curcuminoids was performed by injecting single-standard solutions of each curcuminoid (50 μg/mL) in a mix of water and ethanol (50:50, vol%) prepared and stored in dark.

### UHPLC-QTOF/MS analysis

A fast method was used to confirm the identification of major compounds in the turmeric extract. Briefly, 2 μL of the extract was injected onto a Waters Acquity UPLC BEH-C18 column (100 mm × 2.1 mm, 1.7 μm; Waters Corporation, Milford, MA) using an ACQUITY UPLC® system (Waters Corporation, Milford, MA). The mobile phase consisted of (A) H_2_O and (B) acetonitrile, both containing 0.1% (*v*/v) formic acid. The column temperature was 40 °C and the flow rate 400 μL/min. Gradient elution was used starting at 40% B, then increasing from 40 to 70% B over 0 to 3 min, 70 to 95% B over 3 to 3.5 min, and kept at 95% B for 1 min, and finally returned to initial conditions, for 2 min. MS was performed on a Xevo™ G2 quadrupole time of flight (QTOF) (Waters MS Technologies, Manchester, UK). A mass spectrometer was scanning from 100 to 1000 m/z, the cone voltage was set to 35 V and the capillary voltage was set to 2.5 V in negative ESI mode. The desolvation gas flow rate was 600 L/h at a temperature of 500 °C and the cone gas flow rate was 20 L/h. The source temperature was 120 °C.

### Final hyphenated system operating parameters

Milli-Q water was pre-heated to 70 °C before being pumped as the extraction solvent. The extraction vessel was heated inside the GC oven to the set temperature and maintained at the temperature for 1 min after the sample was loaded. The dynamic extraction was started right afterwards with water pumped at a specific flow rate for the whole experiment. The make-up ethanol flow was maintained at 0.1 mL/min for all experiments. Two temperatures, 90 and 140 °C, and two flow rates, 0.5 and 0.8 mL/min, were used in the kinetic study. Each experiment was repeated three times. The column temperature was set at 33 °C. The flow rate was set at 3.2 mL/min. The mobile phase consisted of A, water (0.3% acetic acid) and B, acetonitrile (0.3% acetic acid). The gradient started with 50% B and then ramp up to 56% in 0.5 min. Then, acetonitrile increased to 95% in 0.3 min. The gradient was maintained at 95% for 0.15 min before rapidly decreasing to the starting composition for re-equilibration for 0.25 min. Each analysis lasted 1.2 min in total. Calibration curves of the three curcuminoids were obtained by injecting standard solutions in a mix of water and ethanol (50:50, vol%) of a series of concentrations (1 μg/mL to 333 μg/mL) using the same UHPLC in the hyphenated system under the same optimised chromatographic conditions listed above.

## Results and discussion

The root of the turmeric (*Curcuma longa* L.) plant is widely used in food, traditional medicine and textile colourants in south Asia [16]. The principal components of turmeric are curcumin and its demethoxy and bisdemethoxy derivatives (see Electronic Supplementary Material (ESM) Fig. S1), which have been proven to exhibit various beneficial health and medicinal effects [17, 18]. The use of hot water or pressurised hot water as extraction solvent has been widely applied in many extraction applications including the extraction of curcuminoids from turmeric [16, 19–21]. In the current study, PHWE of three major curcuminoids from a commercial turmeric power under different extraction conditions was performed as a proof of concept of the new extraction–chromatography system coupled using parallel sampling.

### Tuning of the liquid chromatography separation

**Fig. 2:**
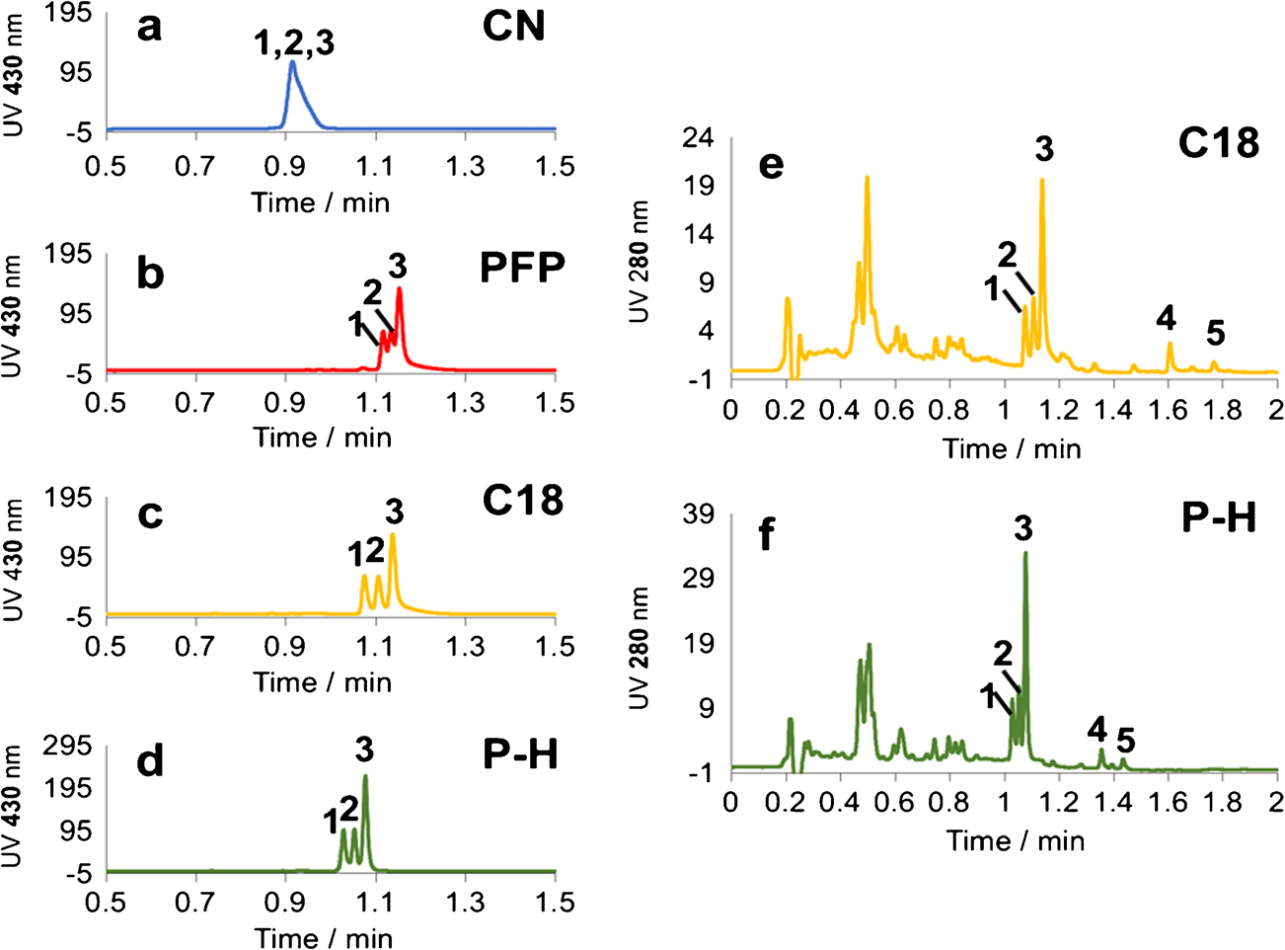
LC column screening results on four columns and two wavelengths. **a**–**d** Screening chromatograms for different columns monitored at 430 nm. **e**, **f** Screening chromatograms for C18 and P-H columns monitored at 280 nm (for chromatographic conditions, see the “Materials and methods” section). Peaks 1, 2 and 3 are bisdemethoxycurcumin, demethoxycurcumin and curcumin respectively. Peaks 4 and 5 are two phospholipids

Figure 2 (left side) shows column screening chromatograms of a turmeric PHWE extract detected at 430 nm. The CN column gave apparently the worst performance with a wide peak and hardly any separation of the three curcuminoids. Among the other three screened columns, the P-H and C18 columns exhibited similar resolution of the targeted compounds (Rs = 0.95, 0.90 and 0.97, 0.93 for the two pairs of peaks separated on P-H and C18 columns, respectively). However, the P-H column provided narrower peak width and better peak shape. Another aspect that should be taken into consideration in the selection of the UHPLC column is the analysis time. Chromatograms at 280 nm revealed that a wider profile of compounds was extracted (Fig. 2, right side). Peaks eluting before the curcuminoids were likely composed of a variety of phenolic compounds, flavonoids and their glycosides [22]. Two major peaks that eluted late in the chromatograms (peaks 4 and 5) consisted of mainly two phospholipids, as confirmed by performing UHPLC-QTOF/MS analysis. It is obvious that for these relatively less polar compounds, the retention was stronger on the C18 column than on the P-H column. Thus, the usage of the C18 column would require longer analysis time in order to avoid un-eluted compounds interfere with the analysis of the next collected extract fraction, which is similar to the wrap-around issues in two-dimensional liquid chromatography [23]. The P-H column was therefore chosen for optimization as shorter analysis time allows higher sampling frequency, which adds more details into the final extraction profiles generated. With the final optimised UHPLC method, the analysis time was shortened to 1.2 min. The limits of detection and quantification (3 and 10 times signal-to-noise ratio) were 25 ng/mL and 100 ng/mL respectively for the three curcuminoids. The absorption behaviour of the curcuminoids was linear from approximately 100 ng/mL to 400 μg/mL.

### Dynamic extraction and analysis of curcuminoids from turmeric

**Fig. 3:**
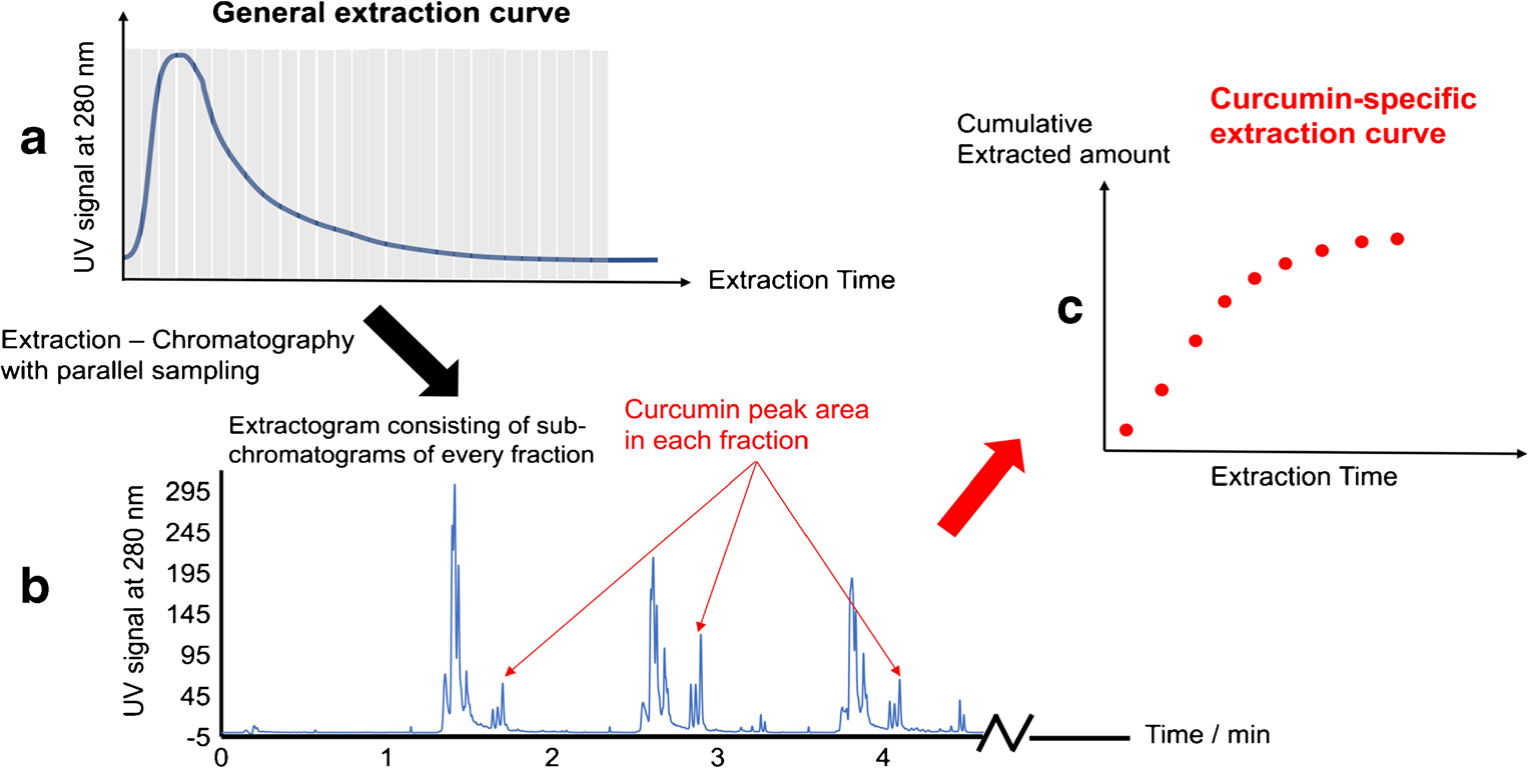
**a**–**c** A general scheme demonstrating how UHPLC chromatograms of all fractions in a PHWE-UHPLC run are used to generate a compound-specific extraction curve

If an extraction process is coupled with an on-line UV detector, a general extraction curve can usually be obtained showing the UV absorption of the eluting extract at different time points (Fig. 3a). However, information regarding specific compounds can be very difficult to acquire from the general extraction curve. With the system reported in this work, the PHWE extract was divided into a series of short fractions and each fraction was sequentially collected and analysed by UHPLC. The hyphenated system can thus produce a series of continual short LC chromatograms monitoring the whole process (Fig. 3b). These chromatograms can be used for three main purposes: (1) to determine the content of compounds in the extract at a certain time; (2) to generate a compound-specific extraction yield vs. extraction time curve in order to understand the kinetics of the extraction (Fig. 3c); and (3) to monitor the occurrence of possible degradation or other reactions taking place during the extraction, i.e. extraction bias.

**Fig. 4:**
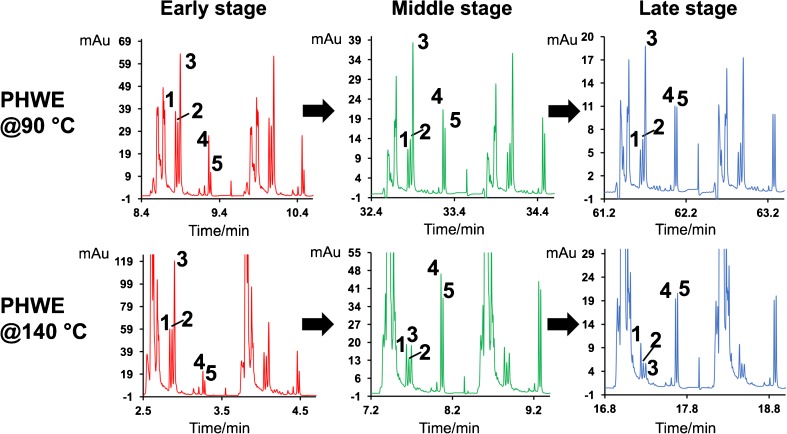
Compound profiles in extracts at different extraction times for two different extraction temperatures, showing two consecutive fractions per chromatogram. Peaks 1, 2 and 3 are bisdemethoxycurcumin, demethoxycurcumin and curcumin respectively. Peaks 4 and 5 are two phospholipids

Figure 4 shows the specific profiles of major compounds in the extract during different stages of the extraction processes under different temperatures. This type of data can be used for several purposes, for instance to quantify specific molecular species in complex sample mixtures; to obtain extraction kinetic data for selected compounds to explore selectivity evolvement during the extraction; and to assess extraction bias in terms of molecular degradation/reaction. For example, it can be noted from Fig. 4 that when the extraction was carried out at 90 °C, the relative amount of extracted curcumin (peak area) in comparison with the other two curcumin derivatives kept increasing. Interestingly, a completely opposite tendency was observed when the extraction temperature was set at 140 °C; the ratio between extracted curcumin and the other two derivatives kept decreasing rapidly during the whole extraction process. This could be the results of compound degradation as it has long been known that curcuminoids, especially curcumin, have poor thermal stability in an aqueous environment [24, 25].

**Fig. 5:**
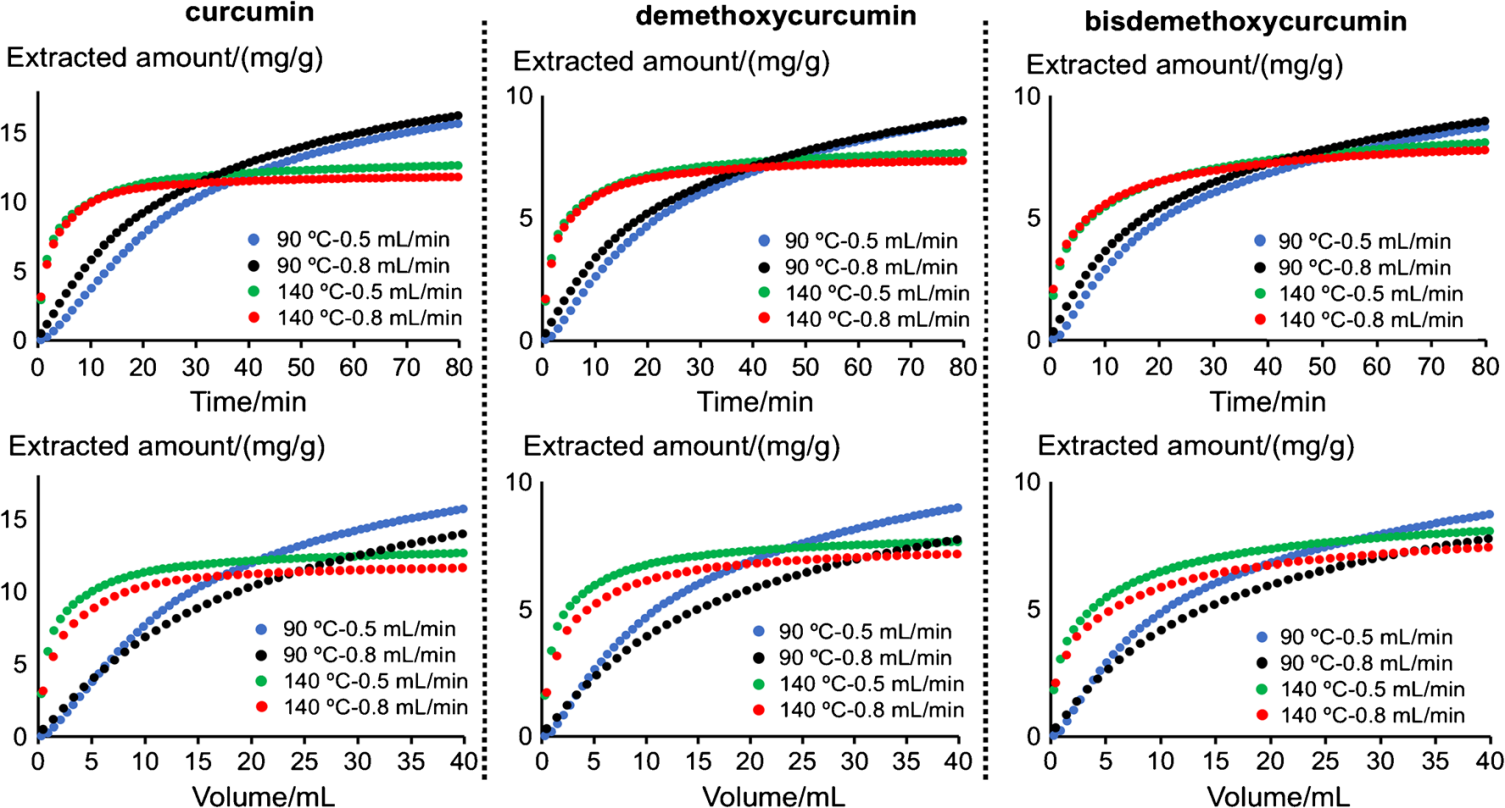
Extraction curves for three curcuminoids under different conditions (see the “Materials and methods” section for UHPLC conditions)

As illustrated in Fig. 3, extraction curves can be constructed for specific compounds. Figure 5 shows extraction curves for the three investigated curcuminoids, obtained at different extraction temperatures (90 and 140 °C) and flow rates (0.5 and 0.8 mL/min). These curves can be used to achieve information about the initial extraction rate, the overall progress of the extraction, as well as the final extractable amount given a certain extraction condition. The progression of the extraction can be described as a function of extraction time or solvent volume. For instance, as shown in Fig. 5 (lower part) where the extracted amount is expressed as a function of solvent volume, it is clear that the initial stage of the extractions conducted at 90 °C gives overlapping extraction curves, i.e. the extraction rate is mainly solubility-controlled under this condition. After ca. 9–14 mL of extraction solvent used, the extraction curves deviate, and the extraction rate is more controlled by mass transfer.

For the higher extraction temperature (140 °C), the initial extraction rate is significantly faster for all three compounds compared with extractions conducted at 90 °C. Also, the extraction curves at 140 °C were close to levelling out at 80 min. However, it can be observed from the trends of the extraction curves that the final extracted amount would be lower than that using the lower temperature. This is true even though the extraction at the lower temperature is far from being a complete extraction at 80 min. A higher extraction temperature enables both higher solubility and faster mass transfer; however, as shown by the overlapping curves when plotted vs. extraction time, the extraction is mainly mass transfer limited. Figure 5 also shows that the downside of a higher extraction temperature like 140 °C is thermal degradation, as clearly seen for all three curcuminoids. This is in accordance with a previous study on static extraction [16]. Moreover, a comparison with the static extraction study also revealed that the consumption of solvent could be reduced and extraction speed accelerated if some extraction parameters are optimised, such as particle size of turmeric and pre-treatment of the powder.

Obtained results can also be plotted as extractograms (contour plots), exemplified with PHWE at 90 °C and 140 °C with 0.5 mL/min (see ESM, Figs. S2 to S4). Such figures can be useful to illustrate extractability of compounds under different extraction conditions.

We propose that the hyphenated dynamic extraction–chromatography system using parallel sampling described in this study enables the quantification of important factors in extraction such as initial extraction rate, solubility vs. mass transfer–controlled rate-limiting stages of the extraction, as well as potential degradation of specific compounds during the extraction, in addition to quantification of total extracted amount (see Table 1 for calculated initial extraction rates of all three curcuminoids under all experimental conditions). Again, it is clear that for the initial extraction rate, constants at the lower temperature are very similar when calculated per solvent volume unit, demonstrating solubility-controlled extraction kinetics, whilst for the higher temperature, the extraction rate constants are similar when calculated per time unit, demonstrating an example of mass transfer–controlled extraction kinetics, in this case also combined with degradation. Here, when plotted per solvent volume unit, an obvious extract dilution takes place with the higher flow rate.

**Table 1:** Initial extraction rate under all experimental conditions, calculated both as a function of time and as a function of solvent volume. Data obtained in the range where the extraction curves have a *R*^2^ value higher than 0.99

	Initial rate as a function of time (mg/(g*min))			
	90 °C 0.5 mL/min	90 °C 0.8 mL/min	140 °C 0.5 mL/min	140 °C 0.8 mL/min
Curcumin	0.36	0.59	1.75	1.61
Demethoxycurcumin	0.26	0.38	1.13	1.03
Bisdemethoxycurcumin	0.29	0.41	0.81	0.76
	Initial rate as a function of solvent volume (mg/(g*mL))			
	90 °C 0.5 mL/min	90 °C 0.8 mL/min	140 °C 0.5 mL/min	140 °C 0.8 mL/min
Curcumin	0.72	0.73	3.50	2.01
Demethoxycurcumin	0.52	0.48	2.26	1.29
Bisdemethoxycurcumin	0.58	0.52	1.62	0.95

Compilation of the data (as shown in Fig. 5) requires the retention time of the analyte peaks to be highly repeatable. The repeatability and reproducibility of the system were accessed with curcumin peaks from 10 randomly selected LC runs in 1 day and curcumin peaks from six randomly selected LC runs in six different days. The within-day relative standard deviation of retention time was determined to be 0.34% and the between-day relative standard deviation of retention time was determined to be 1.2%.

### Comparison with traditional off-line analysis

**Fig. 6:**
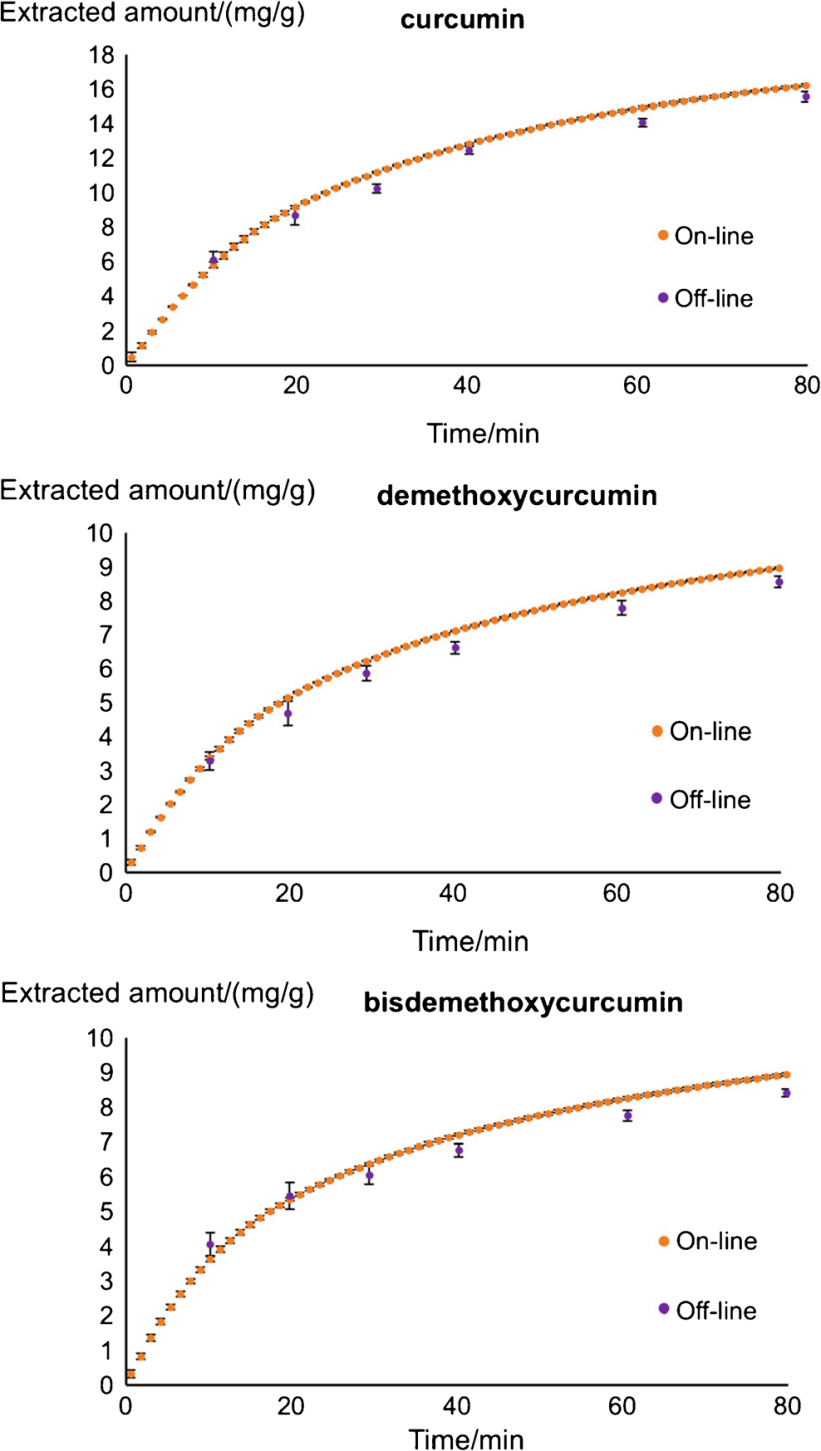
Comparison between the proposed on-line approach and the off-line approach. Extraction was performed under 90 °C with 0.8 mL/min as extraction flow rate (see the “Materials and methods” section for UHPLC conditions). Error bar represents standard deviation of a specific point obtained with 3 repeated experiments

In order to verify the reliability of the extraction curves obtained with the developed on-line system, extractions were performed with the same PHWE system. The extract was collected periodically in fractions, which were followed by off-line analysis using the same UHPLC system included in the hyphenated set-up. As can be seen in Fig. 6, the traditional off-line analysis yielded similar extraction curves as the corresponding curves generated from the on-line system. Meanwhile, much more detailed information regarding the compound extracted during the extraction process can be acquired using the on-line system with all the chromatographic analysis completed along with the extraction. Even though data processing of the large number of short chromatograms obtained can be time-consuming, the on-line system eliminated the extra sample preparation and chromatographic analysis time in the off-line approach.

### Limitations with the system and future aspects

In a traditional 2D-LC system, samples are commonly filtered before the injection and guard columns are usually installed before the first-dimension column. However, in a similar system coupling extraction and chromatography presented in this work, the extract enters the LC column directly. Under this circumstance, guard columns should instead be used before the column to filter out particles and contaminants, which in the meantime increases the gradient delay volume and elution time of strongly retained compounds. For samples containing relatively high numbers of targeted compounds to be extracted, the analysis time should be inevitably prolonged to achieve a decent separation. This leads to lower sampling frequency of the interface and possible loss of information.

In order to improve and apply the parallel sampling interfacing concept to other types of extractions, future work should be focused on two aspects: (1) The interface design has to be modified according to the specific extraction technique. For example, sampling loops could be replaced by solid phase traps to effectively capture and concentrate the compounds from the extraction process. (2) Apply mass spectrometry detection after chromatography to further increase the resolving power and enable compound identification.

## Conclusions

Dynamic pressurised hot water extraction was coupled on-line with ultra-high-performance liquid chromatography using a parallel sampling interface and applied as a proof of concept to a compound-specific kinetic study. The profiles of compounds extracted at different times of the extraction were monitored. Extraction curves for the curcuminoids demonstrated that solubility was the main initial controlling factor for extraction at 90 °C, whilst extractions conducted at 140 °C enabled faster extraction rates although the extracted amount was hampered by severe thermal degradation. This study showed for the first time that coupling extraction with chromatography with parallel sampling can be very useful for the study of the extraction behaviours of compounds or compound classes in a dynamic extraction process. The obtained results were also confirmed by an off-line method.

## Electronic supplementary material

ESM 1 (PDF 2448 kb)
