# Supplementary material for: Dynamic extraction coupled on-line to liquid chromatography with a parallel sampling interface—a proof of concept for monitoring extraction kinetics
Source: Anal Bioanal Chem. 2019 May 6;411(16):3675–83. doi: 10.1007/s00216-019-01849-4 (PMC6571099; doi:10.1007/s00216-019-01849-4)
Supplement: Supplementary file 1 — (PDF 2448 kb) [file 216_2019_1849_MOESM1_ESM.pdf]

Analytical and Bioanalytical Chemistry

Electronic Supplementary Material

Dynamic extraction coupled on-line to liquid chromatography with a parallel sampling interface—a proof of concept for monitoring extraction kinetics

Mingzhe Sun, Said Al-hamimi, Margareta Sandahl, Charlotta Turner

1 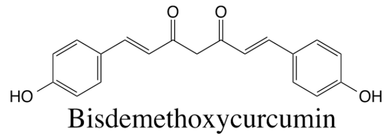

Bisdemethoxycurcumin

2 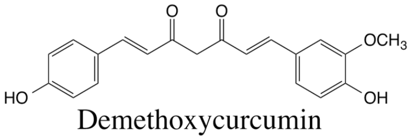

Demethoxycurcumin

3 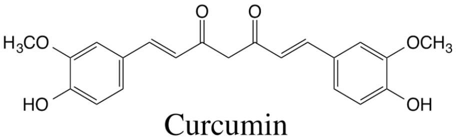

Curcumin

Fig S1 The three major curcuminoids in turmeric

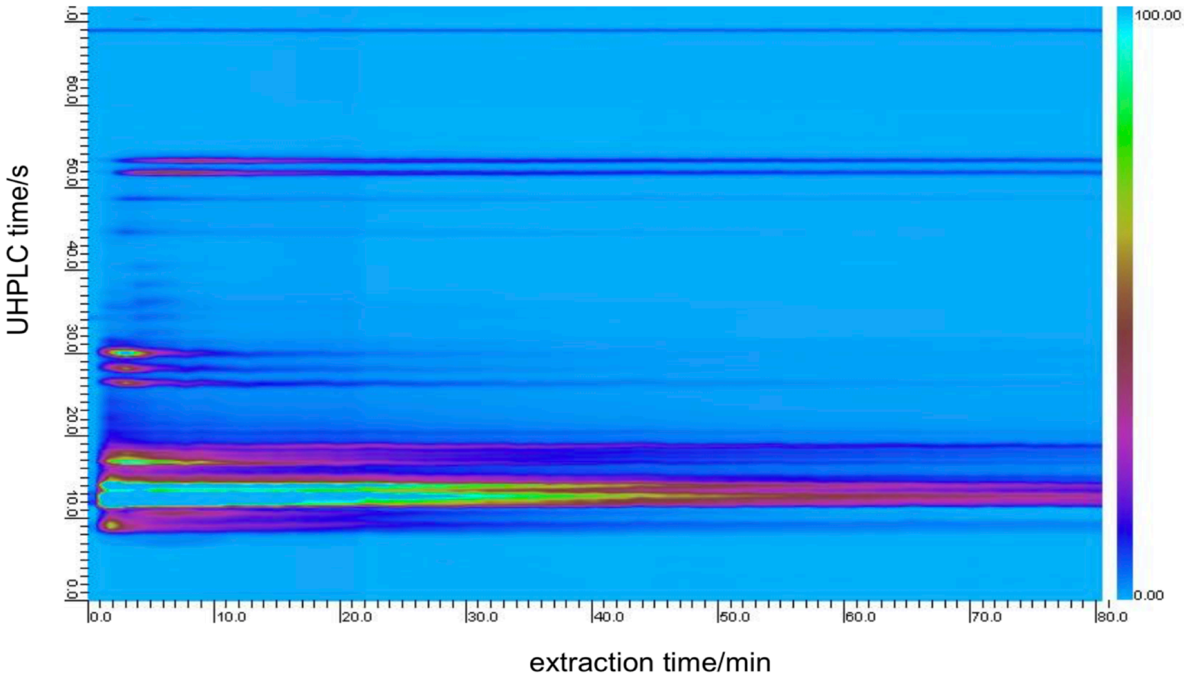

Fig S2 Extractogram at 280 nm of PHWE-UHPLC, PHWE Conditions: 140 °C and 0.5 mL/min

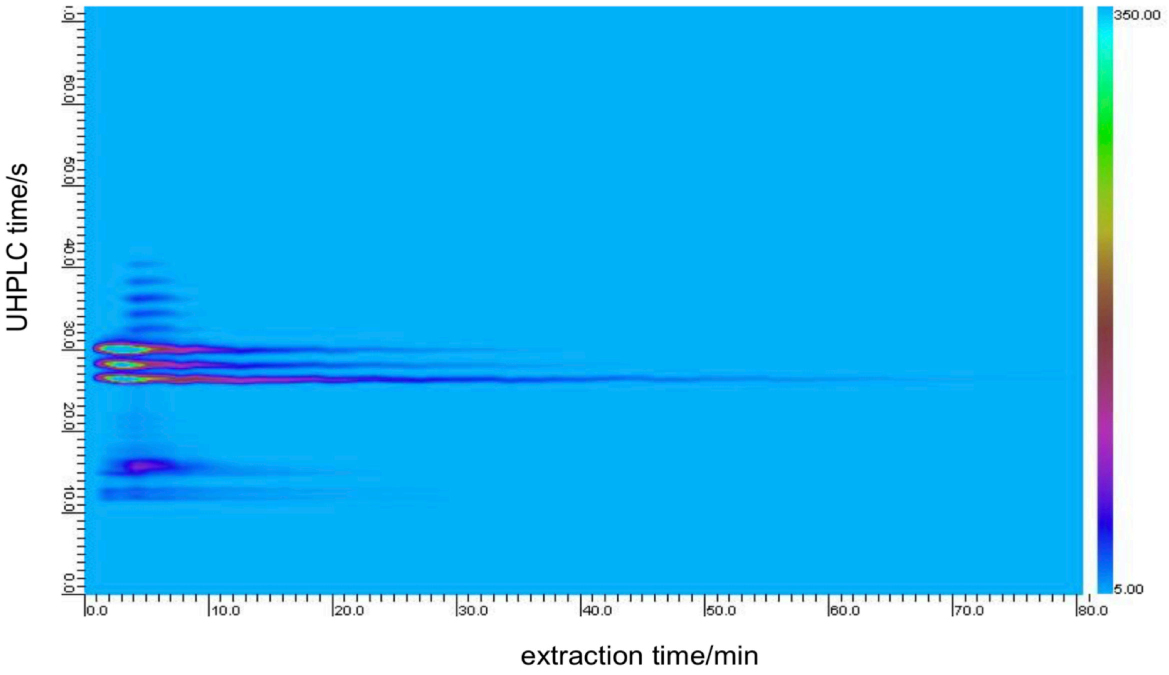

Fig S3 Extractogram at 430 nm of PHWE-UHPLC, PHWE Conditions: 140 °C and 0.5 mL/min

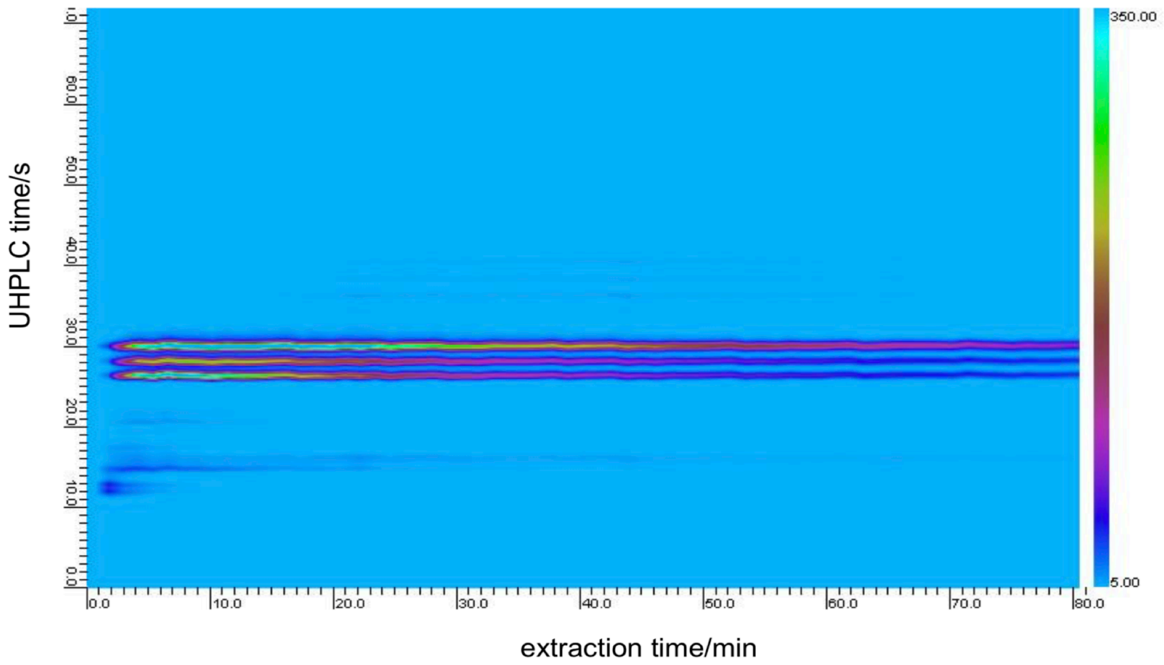

Fig S4 Extractogram at 430 nm of PHWE-UHPLC, PHWE Conditions: 90 °C and 0.5 mL/min
